# Correction: Interventions that improve maternity care for immigrant women in the UK: protocol for a narrative synthesis systematic review

**DOI:** 10.1136/bmjopen-2017-016988corr1

**Published:** 2017-09-18

Higginbottom GMA, Evans C, Morgan M*, et al. *Interventions that improve maternity care for immigrant women in the UK: protocol for a narrative synthesis systematic review. *BMJ Open* 2017;7:e016988. doi: 10.1136/bmjopen-2017-016988

The Corresponding author for this paper is:

Gina Marie Awoko Higginbottom

The Mary Seacole Professor of Ethnicity and Community Health, Faculty of Medicine & Health Sciences

Queen’s Medical Centre

Nottingham

NG7 2UH

UK

Tel: 0115 82 30991

e-mail: Gina.Higginbottom@nottingham.ac.uk

