# Correction: *Interventions that improve maternity care for immigrant women in the UK: protocol for a narrative synthesis systematic review*

**DOI:** 10.1136/bmjopen-2017-016988corr2

**Published:** 2018-01-16

Higginbottom GMA, Evans C, Morgan M, *et al*. Interventions that improve maternity care for immigrant women in the UK: protocol for a narrative synthesis systematic review. *BMJ Open* 2017;7:e016988. doi: 10.1136/bmjopen-2017-016988

The following Disclaimer statement should have been included in the article:

**Disclaimer** The views expressed are those of the author(s) and not necessarily those of the NHS, the NIHR or the Department of Health.

