# Supplementary material for: Interventions that improve maternity care for immigrant women in the UK: protocol for a narrative synthesis systematic review
Source: BMJ Open. 2017 Jul 12;7(7):e016988. doi: 10.1136/bmjopen-2017-016988 (PMC5726105; doi:10.1136/bmjopen-2017-016988)
Supplement: Supplementary file [file bmjopen-2017-016988supp001.pdf]

Appendices:
Table-I
10/03/2017
Ovid MEDLINE(R) In-Process & Other Non-Indexed Citations and Ovid MEDLINE(R) 1946 to Present

1	exp Maternal Health Services/ or exp Postnatal Care/ or exp Preconception Care/ or exp Prenatal Care/ or exp Perinatal Care/ or exp Infant Care/ or exp Midwifery/ or exp Obstetrics/ or exp Obstetric Nursing/
2	exp maternal welfare/ or exp maternal care/ or exp maternal child health care/ or exp newborn care/ or exp prepregnancy care/
3	exp General Practitioners/ or exp Primary Health Care/ or exp Family Health/ or exp Community Health Nursing/
4	(((maternal or child* or baby or babies or fetus* or fetal* or embryo* or obstetric*) adj3 (health* or nurs* or care or service*)) or (midwif* or midwiv*)).ti,ab.
5	((birth* or matern* or mother* or pregnan* or childbearing or child-bearing or prenatal or pre-natal or postnatal or post-natal or perinatal or peri-natal or preconception or pre-conception or antenatal or ante-natal or postpartum or puerperium) adj3 (health* or nurs* or care or service*)).ti,ab.
6	exp Health Services Accessibility/ or exp Healthcare Disparities/ or exp Health Services/
7	(3 or 6) and (matern* or child* or baby or babies or fetus* or fetal* or embryo* or obstetric* or birth* or mother* or pregnan* or childbearing or child-bearing or prenatal or pre-natal or postnatal or post-natal or perinatal or peri-natal or preconception or pre-conception or antenatal or ante-natal or postpartum or puerperium).ti,ab.
8	1 or 2 or 4 or 5 or 7
9	("use" or access* or utili* or consum* or block* or hurdl* or barrier* or hindr* or hinder* or obstacle* or exclu* or discrimin* or disparit* or disproportion* or inequal* or unequal* or inadequat* or insuffic* or stratif* or limit* or lack* or unreliab* or poor* or poverty* or depriv* or disadvantag* or insecur* or insensit* or status* or entitl* or uninform* or ill-inform* or benefit* or interven* or deliver* or effective* or cost effective*).ti,ab.
10	(3 or 6) and 9
11	Emigrants and Immigrants/ or Refugees/ or "Transients and Migrants"/ or "Emigration and Immigration"/
12	(((established or long-term or "first generation*" or new* or recent* or current*) adj3 (migrant* or migrat* or immigrant* or immigrat* or emigrant* or emigrat* or emigre* or expat* or (ex adj pat*) or transient* or alien*)) or newcomer* or (new adj comer*) or incomer* or (in adj comer*) or ((international or overseas or foreign) adj2 (student* or employee* or worker*))).ti,ab.
13	(refugee* or (asylum adj seek*) or asylee* or (refused adj3 (asylum* or refugee*)) or (displaced adj person*) or exile* or (new adj arrival) or (country adj2 (birth or origin)) or transnational*).ti,ab.
14	(foreigner* or (foreign adj (born or citizen* or national* or origin*)) or (non adj (citizen* or native*)) or ((adoptive or naturali#ed) adj (citizen* or resident*)) or overstay* or trafficked or "spousal migrant*").ti,ab.
15	("non-UK-born" or "born outside the UK" or "length of residence in the UK" or (("not lawful*" or "not legal*" or unlawful* or illegal* or unauthori#ed* or "not authori#ed" or uncertain or insecure or illegal or legal or legitimate* or permit* or visa* or irregular* or refused or undocumented) adj3 (residen* or student* or worker* or employee* or unemployed* or immigrant* or imigrat* or migrant* or migrat*))).ti,ab.
16	exp Ethnic Groups/ or (ethnic* or ethno* or race or racial*).ti,ab.
17	exp african continental ancestry group/ or exp asian continental ancestry group/
18	exp Vulnerable Populations/ or ((vulnerab* or disadvantag* or minorit*) adj3 (individ* or person* or people* or population* or communit* or group*)).ti,ab.

19	("Black and Minority Ethnic" or "Black & Minority ethnic" or BME or BAME or african caribbean* or afro caribbean* or black african* or (west adj (indies or indian*))).ti,ab.
20	(south asia* or afghan* or bangladesh* or bengal* or bhutan* or india* or maldiv* or nepal* or pakistan* or sri lanka*).ti,ab.
21	11 or 12 or 13 or 14 or 15 or 16 or 17 or 18 or 19 or 20
22	8 and 10 and 21

Table-II: Preliminary Screening Tool

**Citation** ______________________________________ **Criteria for selection**	Yes	No	Can't say
1. Publication date 1990 to present			
2. English language			
3. Empirical research and findings			
4. Study participants live in the United Kingdom			
5. Study participants are immigrant women (where there is mixed sample of immigrant and non-immigrant women each paper must have findings specific to immigrant women)			
6. Is related to maternity care/birth centres access or interventions or experiences of maternity			

- ❖ An article needs to meet 1-6 to be screened.

Table-III: National Information Centre on Health Services Research and Health Care Technology (NICHSR). Health Services Research and Health Policy Grey Literature Project: Summary Report 2006

5	4	3	2	1
• Working papers<br>• Testimony<br>• Committee reports<br>• Conference proceedings	• Data evaluations<br>• Foundation reports<br>• Government reports<br>• Grantee Publications<br>• Non-commercially published conf. papers<br>• Reports<br>• Special reports	• Speeches<br>• Annual Reports<br>• Presentations<br>• Grantee Reports<br>• Webcasts<br>• Theses<br>• Technical specifications & standards	• Newsletters<br>• Bibliographies<br>• Bulletins<br>• PowerPoint Presentations<br>• Foundation financial statements	• Pamphlets<br>• Protocols<br>• Guidelines<br>• Poster Sessions<br>• Meeting agendas<br>• Translations

Grey literature items falling in columns three to five will be given consideration for inclusion as these demonstrate relatively higher significance to policy makers and related users of grey literature.
