# Supplementary material for: Interventions that improve maternity care for immigrant women in the UK: protocol for a narrative synthesis systematic review
Source: BMJ Open. 2017 Jul 12;7(7):e016988. doi: 10.1136/bmjopen-2017-016988 (PMC5726105; doi:10.1136/bmjopen-2017-016988)
Supplement: Author's manuscript [file bmjopen-2017-016988.draft_revisions.pdf]

BMJ Open

# Interventions that improve maternity care for immigrant women in the United Kingdom (UK): a narrative synthesis systematic review

| | |
|---|---|
| Journal: | *BMJ Open* |
| Manuscript ID | bmjopen-2017-016988 |
| Article Type: | Protocol |
| Date Submitted by the Author: | 31-Mar-2017 |
| Complete List of Authors: | Higginbottom, Gina; University of Nottingham School of Health Sciences<br>Evans, Catrin; University of Nottingham School of Health Sciences<br>Morgan, Myfanwy; King's College London, Primary Care & Public Health Sciences<br>Bharj , Kuldip; University of Leeds, UK, Faculty of Medicine and Health<br>Eldridge, Jeanette; University of Nottingham School of Health Sciences, Research and Learning Services,<br>Hussain, Basharat; University of Nottingham School of Health Sciences, |
| <b>Primary Subject Heading</b>: | Health services research |
| Secondary Subject Heading: | Obstetrics and gynaecology |
| Keywords: | Immigrant women, Maternity care, United Kingdom, Systematic review, Narrative synthesis |
| | |

SCHOLARONE™
Manuscripts

# Interventions that improve maternity care for immigrant women in the United Kingdom (UK): a narrative synthesis systematic review protocol

*Gina Marie Awoko Higginbottom,[1] Catrin Evans,[2] Myfanwy Morgan,[3] Kuldip Kaur Bharj,[4] Jeanette Eldridge,[5] Basharat Hussain[6]*

**Abstract**

**Introduction:**

A quarter of all births in the UK are to mothers born outside the UK.[1] There is also evidence that immigrant women have higher maternal and infant death rates and of inequalities in the provision and uptake of maternity services. The topic is of great significance to the National Health Service (NHS) because of directives that address inequalities and the changing patterns of migration to the UK.[2] A knowledge synthesis will build a coherent evidence base and understanding of factors generating disparities in accessibility, acceptability, and outcomes during maternity care and assist in improving services. Our main question for the review is "what interventions exist that are specifically focused on improving maternity care for immigrant women in the UK"? Sub-questions include: how do these interventions address inequality?; how do accessibility and acceptability manifest, as important dimensions of access to maternity care services as perceived and experienced by immigrant women?

**Methods and analysis:**

Our systematic review uses a narrative synthesis (NS) approach to identify, appraise and synthesise empirical evidence focused on access and interventions that improve maternity care for immigrant women in the UK. We will review qualitative, quantitative and mixed-methods research evidence. A comprehensive set of literature databases and grey literature resources will be searched for relevant articles published between 01 January 1990 and 30 June 2017. The searching and retrieval of results will be completed between February-June 2017. The inclusion criteria is given in the appendix (Table-1). The review will used Gulliford et al.[3] theoretical framework on access in healthcare context.

To ensure robustness of the NS, the methodological quality of key literature will be appraised using the Center for Evidence Based Management tools [4] and review confidence with CERQual.[5] We will use independent blind review method. Two reviewers will independently screen articles, extract relevant data. We will synthesise evidence exploring relationships within and between studies using a range of tools.



For peer review only - http://bmjopen.bmj.com/site/about/guidelines.xhtml

**Dissemination:**

Dissemination plan include: an e-workshop for policy makers, collaborative practitioner workshops, YouTube video and APP, scientific papers and conference presentations.

**Study registration number:** PROSPERO 2015:CRD42015023605

| **Strengths and limitations of this study** |
|---|
| • Our systematic review uses a narrative synthesis (NS) approach to identify, appraise and synthesise reports on empirical research focused on access and interventions that improve maternity care for immigrant women.<br>• A NS a) facilitates understanding and acknowledges the broader influences of theoretical and contextual variables such as ethnicity, socioeconomic status, and geographical location; b) enables understanding of the shaping of differences between reported outcomes as a result of differing study designs and childbearing populations; c) provides results that enable the development and implementation of maternity services and interventions.<br>• We will review qualitative, quantitative and mixed-methods research evidence.<br>• The review will use Gulliford et al.[3] theoretical framework on access in healthcare context.<br>• This is not a meta-synthesis thus limiting our access to the raw data. |

**INTRODUCTION**

The UK is in a period of super-diversity [6] with more diverse populations accessing maternity care.[6-9] Super-diversity "is distinguished by a dynamic interplay of variables among an increased number of new, small and scattered, multiple- origin, transnationally connected, socio-economically differentiated and legally stratified immigrants" [10 p:1024] who started arriving in the UK after 1990. It has been argued that current level of super-diversity in the UK has resulted in a huge challenge for understanding and meeting individualised healthcare needs.[10]

Facilitating the provision of appropriate health care for immigrant populations in the UK is critical for maximizing health potential and well-being.[7 8 11-13] Without culturally appropriate and safe care delivery, a negative trajectory of events may occur that range from simple miscommunication to life-threatening incidents with serious consequences in maternity.[14] For example, immigrant women are over-represented in mortality statistics particularly in maternal and perinatal mortality.[15 16] Whilst recent reviews have focused on specific maternity care aspects [11-13] they have ignored a comprehensive conceptualisation of access as opposed to Gulliford et al,[3] and failed to embrace current



For peer review only - http://bmjopen.bmj.com/site/about/guidelines.xhtml

super-diversity.[6] Reconfiguration and design of maternity care in the UK requires integration of all these aspects.

Our review uses Gulliford theory of access.[3] This framework of access is related to healthcare and has four dimensions (see box below).

i. Service availability
ii. Utilisation of services and barriers to access, which includes personal, financial and organisational barriers
iii. Relevance, effectiveness and access
iv. Equity and access.

These four dimensions are extremely pertinent in respect of providing a theoretical lens in order to explore the experience of immigrant women. We use this [3] theory because primacy is given to the notion of equity of access as the most significant dimension. The NHS seeks to provide a universal and egalitarian service consequently this might be the most profound and significant dimension of access. Our review provides the opportunity for a nuanced and comprehensive exploration of the four facets of access.

Gulliford theoretical framework on access interfaces with NICE[17] research recommendations on access and models of service provision. NICE regards some immigrant women as having complex social factors. It notes that pregnant women, who are recent migrants, asylum seekers or refugees, or who have difficulty reading or speaking English, may not make full use of antenatal care services. This may be because of unfamiliarity with the health service or because they find it hard to communicate with healthcare staff. The NICE guidance suggests that *"healthcare professionals should help support these women's uptake of antenatal care services by, using a varied of means to communicate with women, telling women about antenatal care services and how to use them, and undertaking training in the specific needs of women in these groups" (p.16).* The guidance further recommends that healthcare staff be given specific training in the meeting the needs of the group.

Our aim is to undertake a narrative synthesis of a wide range of empirical literature including synthesis of grey literature to provide stakeholders with perspectives on access and maternity care interventions (NHS & non NHS) directed at immigrant women in the UK.

Our objectives for this synthesis include:



For peer review only - http://bmjopen.bmj.com/site/about/guidelines.xhtml

- To identify, appraise and synthesize qualitative, quantitative and mixed-methods empirical studies on the topic,
- To identify, appraise and synthesize grey literature and non-empirical reports,
- To identify additional knowledge users and mechanisms of knowledge transfer (KT)
- To share our findings through strategic end-of-grant KT.

Ultimately, we will establish the current knowledge base and generate important recommendations for future policy and practice/programming thus mapping out pathways to health equity.

To reach our aim we are using a project advisory group (PAG), initiated during the establishment of the review questions and early planning for dissemination, and as planned for the entire project duration. The group will be active partner in the research process and engage in all steps in the review.

**METHODS AND ANALYSIS**

We will follow a narrative synthesis approach to systematic review. According to Popay et al NS is "an approach to the systematic review and synthesis of findings from multiple studies that relies primarily on the use of words and text to summarise and explain the findings of the synthesis".[18p.5] The emphasis is on an interpretive synthesis of the narrative findings of research rather than on a meta-analysis of the data. NS will allow us to embrace cross-disciplinary and methodologically pluralistic research to map access and interventions in maternity care. The major findings of this synthesis will then be used to explain how and why maternity care interventions focused on immigrant women have been implemented and how these interventions address access and inequalities experienced by immigrant women. Our preliminary scoping review (devised by a research librarian) has helped identify a range of quantitative and qualitative studies focused on access and interventions which enabled us to determine that a synthesis does not yet exist with a specific focus on our review question.

NS comprises four elements: a) Development of a theory of how the intervention works, why it works, and for whom b) Development of a preliminary synthesis of the findings of the included studies c) Exploration of the relationships in the data d) Assessment of the robustness of the synthesis. These elements are not exclusive and the synthesis takes an iterative approach. In each element, a variety of tools and techniques may be used depending on the nature of the research evidence.



For peer review only - http://bmjopen.bmj.com/site/about/guidelines.xhtml

## Eligibility criteria

### Study characteristics

This review will review qualitative, quantitative and mixed methods research evidence. Therefore, no study will be excluded based on its type. All research evidences published in English language from 1990 to present will be eligible for inclusion in the review. We are focusing 1990 as the immigration patterns in the UK changed 1990 onward. Prior to 1990, immigration to the UK was predominately from India, Pakistan, Bangladesh, and Caribbean. The review will include peer reviewed and grey literature.

### Population

We will review empirical and grey literature that report on maternity care for immigrant women in the UK. We define an immigrant as a person who is born outside the UK, has foreign citizenship, and have moved to the UK to stay temporarily (at least a year) or has intention to settle for the long-term.[19] Therefore, economic migrants (both skilled and unskilled), refugees, asylum seekers and students population will be included.[20]

### Intervention

By intervention we mean "a combination of program elements or strategies designed to produce behavior changes or improve health status among individuals or an entire population".[21] The interventions that we plan to review are those that immigrants participate in during the antenatal, intra-partum or postnatal period in healthcare or social or community setting. Therefore only interventions relevant to pregnancy and the postpartum period (up to 12 months post-delivery) will be reviewed. Interventions must be specifically focused on immigrant women and those aimed at the general population will be excluded.

### Context

Maternity health services related interventions that are offered in the United Kingdom. Interventions focused on immigrants in any setting i.e. hospital and community will be included.

### Outcomes

This synthesis seeks to address gaps in knowledge and generate new interpretations of research findings and other literature for translation into improvements to maternity services provision throughout the UK for immigrant women. The project does not directly evaluate interventions/impacts on service delivery but will provide an important basis to guide such developments and ensure their acceptability and effectiveness for immigrant women.



For peer review only - http://bmjopen.bmj.com/site/about/guidelines.xhtml

**Data sources and search strategy**

The following literature databases will be searched for relevant articles published between 01 January 1990 and 30 June 2017; the searching and retrieval of results will be completed between February-June 2017. The quoted date range for each database and preferred host system are recorded below:

- Medline (1946 – present, Ovid),
- Embase (1980 – present, Ovid),
- PsycINFO (1806 – present, Ovid),
- HMIC (1979 – latest monthly update, Ovid),
- MIDIRS (1971 – latest monthly update, Ovid),
- CINAHL (to present, EBSCOHost),
- ASSIA (1976 – present, ProQuest),
- POPline (http://www.popline.org),
- Web of Science (1900 – present),
- Scopus (1970 – present)

An experienced research librarian will construct the detailed search strategies for each database and conduct the searches after review by the entire team. The Ovid MEDLINE search strategy will be adapted for each database so that relevant controlled vocabulary, searching techniques, and keywords are used (attached). Reference lists of included studies will be reviewed for relevant citations and additional hand-searches will be undertaken in major relevant journal e.g. J Immigr Health) and as published by topical research groups. BH and JE will implement the search strategy independently (independent double screening), and meet to review any discrepancies or dissonance. Ambiguous papers will be reviewed by the entire team.

The search for grey literature will include thesis repositories (e.g. ProQuest Dissertations & Theses), Google Scholar, internet-based searches, citation searches, clinical trials registers, research funders' listings of research projects, reviews of reference lists. Grey literature will be screened for relevance and categorised using the National Information Centre on Health Services Research system.[22]

**Data management**

The review will use Endnote Bibliographic management software to manage the searches. All search hits will be directly imported into the Endnote and duplicates will be removed by the two reviewers independently.



For peer review only - http://bmjopen.bmj.com/site/about/guidelines.xhtml

## Selection process

We will use the PRISMA flow chart (fig.1) to document the review steps.[23] We will employ a three-stage process: (I) screening; (II) preliminary categorization (III) retrieval, final selection and final categorization, employing independent double screening. The screening of peer reviewed articles include screening of titles, screening of abstracts with the attached screening tool given in the appendix (Table-I). This tool will be piloted by two reviewers with the first 10% of the database 'hits'.

Grey literature items will be screened for relevance and categorized using the categories of grey literature established by the National Information Centre on Health Services Research and Health Care Technology at the National Library of Medicine.[22]

**Fig.1 Review stages[24]**

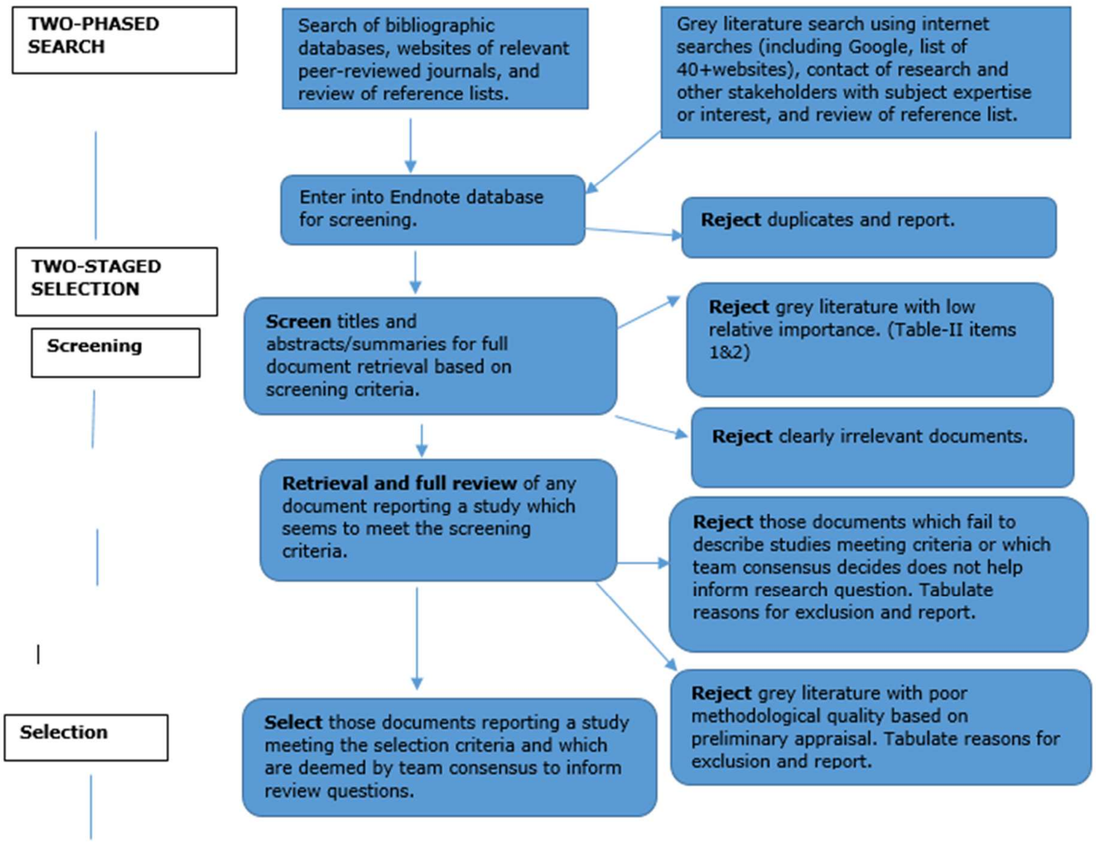


The two reviewers will independently extract data from each included study and any disagreement will be resolved through discussion, if needed involving the third reviewer to reach consensus. Data will be sought on variable such as which migrant group(s) the intervention(s) is focused, length of migration, type of intervention, setting of



For peer review only - http://bmjopen.bmj.com/site/about/guidelines.xhtml

intervention (Hospital/community), duration of intervention, intervention making agency (NHS, social care, voluntary group).

Four researchers experienced in numerous forms of systematic reviewer having undertaken formal training and conducted subsequent funded reviews [24-26] will be involved in data extraction (GH, CE, KB, BH). A relevancy appraisal will be undertaken by first reviewing the title and abstract (GH,CE,KB,BH) and then retrieving potentially relevant articles for further assessment to meet our inclusion criteria and with entire team deciding final inclusion. The methodological quality of key literature will be appraised using the Center for Evidence Based (CEBMa) Management tools (e.g. critical appraisal of a survey, critical appraisal of a qualitative study, critical appraisal of a case study)[4] and for assessment of confidence in the NS review findings CERQUAL.[5] We are using critical appraisal tools of CEBMa as it has a full range of tools as opposed to Critical Appraisal Skills Programme (CASP)e.g. CASP does not have a tool for survey research.

**Missing data**
Attempts will be made to contact study authors via email to obtain clarification if data are incomplete in the study report and we will allow a delay of six weeks to receive a response following two email attempts.

**Quality Appraisal** We plan to assess quality and the robustness of the synthesis through following ways:
*I) Weight of evidence*: Popay et al. [18] identifies the weight of evidence approach as a tool that can be used to assess the robustness of the synthesis results.
*II) Critical reflection:* Popay et al.[18] recommend that a summary discussion of the synthesis should be provided and includes the following:
a) Methodology of the synthesis – especially focusing on the limitations and their possible impact on the results,
b) Evidence used, in terms of quality, reliability, validity and generalizability (for quantitative papers), with an emphasis on possible sources of bias (for qualitative papers we will apply Lincoln and Guba's principles of confirmability, transferability, credibility, and dependability,[27]
c) Assumptions made,
d) Discrepancies and uncertainties identified, and how discrepancies were dealt with,
e) Areas where the evidence is weak or non-existent, possible areas for future research, and finally,
f) A discussion of the evidence presented will consider the 'thick' and 'thin' evidence [25] and comment on similarities and/or differences between evidence (GH, MM, CE, KB, BH).



For peer review only - http://bmjopen.bmj.com/site/about/guidelines.xhtml

'Thick' evidence offers adequate detail to achieve an understanding of the phenomenon of our interest, whereas 'thin' evidence is unable to provide appropriate detail to give an understanding of the phenomenon.[18]

**Strategy for data synthesis**

We have planned a narrative (descriptive) synthesis for this review for all types of studies. Patterns emerging from the textual descriptions and cross-literature comparisons will allow us to identify the factors that affect maternity interventions and the implementation of maternity services. These factors will be synthesized into major themes regarding barriers and enablers that shape interventions related to immigrant women and maternity care services. We will use conceptual and thematic analysis using a range of clustering and networking tools. In addition to tabulation, we will use analytical software for further grouping (i.e atlas.ti7 by ATLAS.ti GmbH, Berlin, Germany).

**DISSEMINATION**

A multi-level knowledge translation (KT) plan will be used so that key messages are strategically delivered to key stakeholders. We will facilitate an online workshop with key-decision makers/stakeholders to ensure policy relevant messages and utilise widely accessible technology (social networking, webinars) to maximize impact.

The research team involves individuals who are engaged in community/hospital-based health services with immigrant women. KT will continue through community meetings with families, women's groups, and workshops related to refugee/immigrant health. Health and social care knowledge users will be invited to attend community-based initiatives e.g. seminars/workshops.

Contribution to theory and practice will occur via publication of findings in high-impact international journals, such as J Immigr Minor Health, Sociology of Health and Illness, Journal of Health Services Research and Policy, and Social Science and Medicine and open-access journals (e.g. BMC Pregnancy & Childbirth, BMJ Open Access).

**Author affiliations**

[1]Prof. Gina Marie Awoko Higginbottom
The Mary Seacole Professor of Ethnicity and Community Health
School of Health Sciences
The University of Nottingham
B Floor, South Block Link, Queen's Medical Centre
Nottingham, NG7 2HA
t: +44 (0) 115 82 30991
e-mail: gina.higginbottom@nottingham.ac.uk



For peer review only - http://bmjopen.bmj.com/site/about/guidelines.xhtml

[2]Dr Catrin Evans, Associate Professor
School of Health Sciences, University of Nottingham, UK
A Floor, South Block Link, Queen's Medical Centre
Nottingham, NG7 2HA
e-mail: catrin.evans@nottingham.ac.uk

[3]Professor Myfanwy Morgan
Emeritus and Visiting Professor
Institute of Pharmaceutical Science
King's College London, UK
e-mail: myfanwy.morgan@kcl.ac.uk

[4]Dr Kuldip Kaur Bharj (KB) OBE, Associate Professor in Midwifery,
Faculty of Medicine and Health
School of Healthcare, University of Leeds, UK
e-mail: k.k.bharj@leeds.ac.uk

[5]Jeanette Eldridge
Senior Research Librarian, Medicine & Health Sciences
Research and Learning Services,
University of Nottingham, UK
e-mail: jeanette.eldridge@nottingham.ac.uk

[6]Dr Basharat Hussain **(Corresponding author)**

Senior Research Fellow
School of Health Sciences
The University of Nottingham
B Floor, South Block Link, Queen's Medical Centre
Nottingham, NG7 2HA
e-mail: basharat.hussain@nottingham.ac.uk

**Contributors**:

GH conceptualised the review and drafted the original grant proposal with CE, MM and KB. JE developed and implemented the scoping review strategy. BH drafted the manuscript for publication with input from other team members. All authors approved the final version of the protocol for publication.

**Funding:**

This work is supported by the UK National Institute for Health Research (NIHR) Health Services and Delivery Research Programme by grant number (HS&DR-15/55/03). Along with funding, NIHR also contributed in peer reviewing the funding proposal containing the review protocol. Any amendments (if required) in the protocol would be done in consultation with NIHR.

**Sponsor:** Department of Health (DoH)- NIHR (543808)

**Competing interests**: None.



For peer review only - http://bmjopen.bmj.com/site/about/guidelines.xhtml

## REFERENCES

1. Office of National Statistics. Birth in England and Wales by Parents Country of Birth, 2013. In: Statistics OoN, ed., 2014.
2. Health Do. *Equity and excellence:: liberating the NHS*: The Stationery Office, 2010.
3. Gulliford M, Figueroa-Munoz J, Morgan M, et al. What does' access to health care'mean? Journal of health services research & policy 2002;**7**(3):186-88.
4. Center for Evidence-Based Management. Critical Appraisal Tools & Questionnaires 2017 [Available from: https://www.cebma.org/.
5. Lewin S, Glenton C, Munthe-Kaas H, et al. Using qualitative evidence in decision making for health and social interventions: an approach to assess confidence in findings from qualitative evidence syntheses (GRADE-CERQual). PLoS Med 2015;**12**(10):e1001895.
6. Phillimore J. Migrant maternity in an era of superdiversity: New migrants' access to, and experience of, antenatal care in the West Midlands, UK. Social Science & Medicine 2016;**148**:152-59.
7. Fisher J, Hinchliff S. Immigrant women's perceptions of their maternity care: a review of the literature part 2. The practising midwife 2013;**16**(2):32-34.
8. Shortall C, McMorran J, Taylor K, et al. Experiences of Pregnant Migrant Women receiving Ante/Peri and Postnatal Care in the UK: A Doctors of the World Report on the Experiences of attendees at their London Drop-In Clinic, 2015.
9. Garcia R, Ali N, Papadopoulos C, et al. Specific antenatal interventions for Black, Asian and Minority Ethnic (BAME) pregnant women at high risk of poor birth outcomes in the United Kingdom: a scoping review. BMC pregnancy and childbirth 2015;**15**(1):226.
10. Vertovec S. Super-diversity and its implications. Ethnic and racial studies 2007;**30**(6):1024-54.
11. Coast E, Jones E, Portela A, et al. Maternity care services and culture: a systematic global mapping of interventions. PLoS One 2014;**9**(9):e108130.
12. Oakley L, Gray R, Kurinczuk J, et al. P14 Interventions to increase the early initiation of antenatal care in socially disadvantaged and vulnerable women: a systematic review. Journal of Epidemiology and Community Health 2010;**64**(Suppl 1):A39-A39.
13. Hollowell J, Oakley L, Vigurs C, et al. Increasing the early initiation of antenatal care by Black and Minority Ethnic women in the United Kingdom: a systematic review and mixed methods synthesis of women's views and the literature on intervention effectiveness. Final Report University of Oxford 2012.
14. Higginbottom GM, Safipour J, Yohani S, et al. An ethnographic study of communication challenges in maternity care for immigrant women in rural Alberta. Midwifery 2015;**31**(2):297-304.
15. Knight M, Kurinczuk JJ, Spark P, et al. Inequalities in maternal health: national cohort study of ethnic variation in severe maternal morbidities. BMJ 2009;**338**:b542.
16. Manktelow B, Smith L, Seaton S, et al. MBRRACE-UK Perinatal Mortality Surveillance Report, UK Perinatal Deaths for Births from January to December 2014. The Infant Mortality and Morbidity Studies, Department of Health Sciences, University of Leicester, Leicester 2016.
17. National Institute for Health and Clinical Excellence (NICE). Pregnancy and complex social factors: A model for service provision for pregnant women with complex social factors. In: guidelines N, ed., 2010.
18. Popay J, Roberts H, Sowden A, et al. Guidance on the conduct of narrative synthesis in systematic reviews. A product from the ESRC methods programme Version 2006;**1**:b92.
19. Anderson B, Blinder S. Who counts as a migrant? Definitions and their consequences. Briefing, The Migration Observatory at the University of Oxford 2017.



For peer review only - http://bmjopen.bmj.com/site/about/guidelines.xhtml

20. Urquia ML, Gagnon AJ. Glossary: migration and health. Journal of Epidemiology and Community Health 2011:jech. 2010.109405.
21. Intervention MICA : Building healthy communities. What is an intervention 2017 [Available from: http://health.mo.gov/data/interventionmica/.
22. National Information Center on Health Services Research and Health Care Technology (NICHSR). Health Services Research and Health Policy Grey Literature Project: Summary Report 2006 [Available from: https://www.nlm.nih.gov/nichsr/greylitreport_06.html.
23. Moher D, Liberati A, Tetzlaff J, et al. Preferred reporting items for systematic reviews and meta-analyses: the PRISMA statement. PLoS med 2009;**6**(7):e1000097.
24. Higginbottom GM, Morgan M, Alexandre M, et al. Immigrant women's experiences of maternity-care services in Canada: a systematic review using a narrative synthesis. Syst Rev 2015;**4**(1):13-17.
25. Higginbottom GM, Hadziabdic E, Yohani S, et al. Immigrant women's experience of maternity services in Canada: A meta-ethnography. Midwifery 2014;**30**(5):544-59.
26. Higginbottom G, Bell AS, Arsenault J, et al. An integrative review of experiences of maternity services for immigrant women in Canada. Diversity & Equality in Health and Care 2012.
27. Lincoln YS, Guba EG. *Naturalistic inquiry*: Sage, 1985.

## Appendices

**Table-I: Preliminary Screening Tool**

| **Citation** ________________________________<br>**Criteria for selection** | Yes | No | Can't say |
|---|---|---|---|
| 1. Publication date 1990 to present | | | |
| 2. English language | | | |
| 3. Empirical research and findings | | | |
| 4. Study participants live in the United Kingdom | | | |
| 5. Study participants are immigrant women (where there is mixed sample of immigrant and non-immigrant women each paper must have findings specific to immigrant women) | | | |
| 6. Is related to maternity care access or interventions or experiences of maternity | | | |

- An article needs to meet 1-6 to be screened.

**Table-II: National Information Centre on Health Services Research and Health Care Technology (NICHSR). Health Services Research and Health Policy Grey Literature Project: Summary Report 2006**

| 5 | 4 | 3 | 2 | 1 |
|---|---|---|---|---|
| • Working papers<br>• Testimony | • Data evaluations<br>• Foundation | • Speeches<br>• Annual Reports | • Newsletters<br>• Bibliographies<br>• Bulletins | • Pamphlets<br>• Protocols<br>• Guidelines |



For peer review only - http://bmjopen.bmj.com/site/about/guidelines.xhtml

| | | | | |
|---|---|---|---|---|
| • Committee reports<br>• Conference proceedings | reports<br>• Government reports<br>• Grantee Publications<br>• Non-commercially published conf. capers<br>• Reports<br>• Special reports | • Presentations<br>• Grantee Reports<br>• Webcasts<br>• Theses<br>• Technical specifications & standards | • PowerPoint Presentations<br>• Foundation financial statements | • Poster Sessions<br>• Meeting agendas<br>• Translations |

Grey literature items falling in columns three to five will be given consideration for inclusion as these demonstrate relatively higher significance to policy makers and related users of grey literature.

For peer review only

| | |
|---|---|
| 1 | exp Maternal Health Services/ or exp Postnatal Care/ or exp Preconception Care/ or exp Prenatal Care/ or exp Perinatal Care/ or exp Infant Care/ or exp Midwifery/ or exp Obstetrics/ or exp Obstetric Nursing/ |
| 2 | exp maternal welfare/ or exp maternal care/ or exp maternal child health care/ or exp newborn care/ or exp prepregnancy care/ |
| 3 | exp General Practitioners/ or exp Primary Health Care/ or exp Family Health/ or exp Community Health Nursing/ |
| 4 | (((maternal or child* or baby or babies or fetus* or fetal* or embryo* or obstetric*) adj3 (health* or nurs* or care or service*)) or (midwif* or midwiv*)).ti,ab. |
| 5 | ((birth* or matern* or mother* or pregnan* or childbearing or child-bearing or prenatal or pre-natal or postnatal or post-natal or perinatal or peri-natal or preconception or pre-conception or antenatal or ante-natal or postpartum or puerperium) adj3 (health* or nurs* or care or service*)).ti,ab. |
| 6 | exp Health Services Accessibility/ or exp Healthcare Disparities/ or exp Health Services/ |
| 7 | (3 or 6) and (matern* or child* or baby or babies or fetus* or fetal* or embryo* or obstetric* or birth* or mother* or pregnan* or childbearing or child-bearing or prenatal or pre-natal or postnatal or post-natal or perinatal or peri-natal or preconception or pre-conception or antenatal or ante-natal or postpartum or puerperium).ti,ab. |
| 8 | 1 or 2 or 4 or 5 or 7 |
| 9 | ("use" or access* or utili* or consum* or block* or hurdl* or barrier* or hindr* or hinder* or obstacle* or exclu* or discrimin* or disparit* or disproportion* or inequal* or unequal* or inadequat* or insuffic* or stratif* or limit* or lack* or unreliab* or poor* or poverty* or depriv* or disadvantag* or insecur* or insensit* or status* or entitl* or uninform* or ill-inform* or benefit* or interven* or deliver* or effective* or cost effective*).ti,ab. |
| 10 | (3 or 6) and 9 |
| 11 | Emigrants and Immigrants/ or Refugees/ or "Transients and Migrants"/ or "Emigration and Immigration"/ |
| 12 | (((established or long-term or "first generation*" or new* or recent* or current*) adj3 (migrant* or migrat* or immigrant* or immigrat* or emigrant* or emigrat* or emigre* or expat* or (ex adj pat*) or transient* or alien*)) or newcomer* or (new adj comer*) or incomer* or (in adj comer*) or ((international or overseas or foreign) adj2 (student* or employee* or worker*))).ti,ab. |
| 13 | (refugee* or (asylum adj seek*) or asylee* or (refused adj3 (asylum* or refugee*)) or (displaced adj person*) or exile* or (new adj arrival) or (country adj2 (birth or origin)) or transnational*).ti,ab. |
| 14 | (foreigner* or (foreign adj (born or citizen* or national* or origin*)) or (non adj (citizen* or native*)) or ((adoptive or naturali#ed) adj (citizen* or resident*)) or overstay* or trafficked or "spousal migrant*").ti,ab. |
| 15 | ("non-UK-born" or "born outside the UK" or "length of residence in the UK" or (("not lawful*" or "not legal*" or unlawful* or illegal* or unauthori#ed* or "not authori#ed" or uncertain or insecure or illegal or legal or legitimate* or permit* or visa* or irregular* or refused or undocumented) adj3 (residen* or student* or worker* or employee* or unemployed* or immigrant* or imigrat* or migrant* or migrat*))).ti,ab. |
| 16 | exp Ethnic Groups/ or (ethnic* or ethno* or race or racial*).ti,ab. |
| 17 | exp african continental ancestry group/ or exp asian continental ancestry group/ |
| 18 | exp Vulnerable Populations/ or ((vulnerab* or disadvantag* or minorit*) adj3 (individ* or person* or people* or population* or communit* or group*)).ti,ab. |

For peer review only

| | |
|---|---|
| 19 | ("Black and Minority Ethnic" or "Black & Minority ethnic" or BME or BAME or african caribbean* or afro caribbean* or black african* or (west adj (indies or indian*))).ti,ab. |
| 20 | (south asia* or afghan* or bangladesh* or bengal* or bhutan* or india* or maldiv* or nepal* or pakistan* or sri lanka*).ti,ab. |
| 21 | 11 or 12 or 13 or 14 or 15 or 16 or 17 or 18 or 19 or 20 |
| 22 | 8 and 10 and 21 |
| | |
| 23 | limit 22 to yr="2017 -Current" |
| 24 | limit 22 to yr="2016" |
| 25 | limit 22 to yr="2015" |
| 26 | limit 22 to yr="2014" |
| 27 | limit 22 to yr="2013" |
| 28 | limit 22 to yr="2012" |
| 29 | limit 22 to yr="2011" |
| 30 | limit 22 to yr="2010" |
| 31 | limit 22 to yr="2009" |
| 32 | limit 22 to yr="2008" |
| 33 | limit 22 to yr="2007" |
| 34 | limit 22 to yr="2006" |
| 35 | limit 22 to yr="2005" |
| 36 | limit 22 to yr="2004" |
| 37 | limit 22 to yr="2003" |
| 38 | limit 22 to yr="2002" |
| 39 | limit 22 to yr="2001" |
| 40 | limit 22 to yr="2000" |
| 41 | limit 22 to yr="1999" |
| 42 | limit 22 to yr="1998" |
| 43 | limit 22 to yr="1997" |
| 44 | limit 22 to yr="1996" |
| 45 | limit 22 to yr="1995" |
| 46 | limit 22 to yr="1994" |
| 47 | limit 22 to yr="1993" |
| 48 | limit 22 to yr="1992" |
| 49 | limit 22 to yr="1991" |
| 50 | limit 22 to yr="1990" |
| 51 | 23 or 24 or 25 or 26 or 27 or 28 or 29 or 30 or 31 or 32 or 33 or 34 or 35 or 36 or 37 or 38 or 39 or 40 or 41 or 42 or 43 or 44 or 45 or 46 or 47 or 48 or 49 or 50 |
| 52 | 22 not 51 |

For peer review only

**PRISMA-P (Preferred Reporting Items for Systematic review and Meta-Analysis Protocols) 2015 checklist: recommended items to address in a systematic review protocol***

| Section and topic | Item No | Checklist item |
|---|---|---|
| **ADMINISTRATIVE INFORMATION** | | |
| Title: | | |
| Identification | 1a | Identify the report as a protocol of a systematic review **(01)** |
| Update | 1b | If the protocol is for an update of a previous systematic review, identify as such **(It is new protocol)** |
| Registration | 2 | If registered, provide the name of the registry (such as PROSPERO) and registration number **(PROSPERO 2015:CRD42015023605)** |
| Authors: | | |
| Contact | 3a | Provide name, institutional affiliation, e-mail address of all protocol authors; provide physical mailing address of corresponding author **(9-10)** |
| Contributions | 3b | Describe contributions of protocol authors and identify the guarantor of the review **(10)** |
| Amendments | 4 | If the protocol represents an amendment of a previously completed or published protocol, identify as such and list changes; otherwise, state plan for documenting important protocol amendments **(10)** |
| Support: | | |
| Sources | 5a | Indicate sources of financial or other support for the review **(10)** |
| Sponsor | 5b | Provide name for the review funder and/or sponsor **(10)** |
| Role of sponsor or funder | 5c | Describe roles of funder(s), sponsor(s), and/or institution(s), if any, in developing the protocol **(10)** |
| **INTRODUCTION** | | |
| Rationale | 6 | Describe the rationale for the review in the context of what is already known **(2-3)** |
| Objectives | 7 | Provide an explicit statement of the question(s) the review will address with reference to participants, interventions, comparators, and outcomes (PICO) **(01, 3-4)** |
| **METHODS** | | |
| Eligibility criteria | 8 | Specify the study characteristics (such as PICO, study design, setting, time frame) and report characteristics (such as years considered, language, publication status) to be used as criteria for eligibility for the review **(05)** |
| Information sources | 9 | Describe all intended information sources (such as electronic databases, contact with study authors, trial registers or other grey literature sources) with planned dates of coverage **(6)** |
| Search strategy | 10 | Present draft of search strategy to be used for at least one electronic database, including planned limits, such that it could be repeated (**6 attached** ) |
| Study records: | | |

For peer review only

| | | |
|---|---|---|
| Data management | 11a | Describe the mechanism(s) that will be used to manage records and data throughout the review **(6)** |
| Selection process | 11b | State the process that will be used for selecting studies (such as two independent reviewers) through each phase of the review (that is, screening, eligibility and inclusion in meta-analysis) **(7-8)** |
| Data collection process | 11c | Describe planned method of extracting data from reports (such as piloting forms, done independently, in duplicate), any processes for obtaining and confirming data from investigators **(7-8)** |
| Data items | 12 | List and define all variables for which data will be sought (such as PICO items, funding sources), any pre-planned data assumptions and simplifications **(7-8)** |
| Outcomes and prioritization | 13 | List and define all outcomes for which data will be sought, including prioritization of main and additional outcomes, with rationale **(5)** |
| Risk of bias in individual studies | 14 | Describe anticipated methods for assessing risk of bias of individual studies, including whether this will be done at the outcome or study level, or both; state how this information will be used in data synthesis **(8)** |
| Data synthesis | 15a | Describe criteria under which study data will be quantitatively synthesised **(N/A)** |
| | 15b | If data are appropriate for quantitative synthesis, describe planned summary measures, methods of handling data and methods of combining data from studies, including any planned exploration of consistency (such as $I^2$, Kendall's τ) **(N/A)** |
| | 15c | Describe any proposed additional analyses (such as sensitivity or subgroup analyses, meta-regression) **(N/A)** |
| | 15d | If quantitative synthesis is not appropriate, describe the type of summary planned **(9)** |
| Meta-bias(es) | 16 | Specify any planned assessment of meta-bias(es) (such as publication bias across studies, selective reporting within studies) |
| Confidence in cumulative evidence | 17 | Describe how the strength of the body of evidence will be assessed (such as GRADE) **(8)** |

*** It is strongly recommended that this checklist be read in conjunction with the PRISMA-P Explanation and Elaboration (cite when available) for important clarification on the items. Amendments to a review protocol should be tracked and dated.** **The copyright for PRISMA-P (including checklist) is held by the PRISMA-P Group and is distributed under a Creative Commons Attribution Licence 4.0.**

*From: Shamseer L, Moher D, Clarke M, Ghersi D, Liberati A, Petticrew M, Shekelle P, Stewart L, PRISMA-P Group. Preferred reporting items for systematic review and meta-analysis protocols (PRISMA-P) 2015: elaboration and explanation. BMJ. 2015 Jan 2;349(jan02 1):g7647.*

For peer review only

BMJ Open

# Interventions that improve maternity care for immigrant women in the United Kingdom (UK): protocol for a narrative synthesis systematic review

| | |
|---|---|
| Journal: | *BMJ Open* |
| Manuscript ID | bmjopen-2017-016988.R1 |
| Article Type: | Protocol |
| Date Submitted by the Author: | 05-Jun-2017 |
| Complete List of Authors: | Higginbottom, Gina; University of Nottingham School of Health Sciences<br>Evans, Catrin; University of Nottingham School of Health Sciences<br>Morgan, Myfanwy; King's College London, Primary Care & Public Health Sciences<br>Bharj , Kuldip; University of Leeds, UK, Faculty of Medicine and Health<br>Eldridge, Jeanette; University of Nottingham School of Health Sciences, Research and Learning Services,<br>Hussain, Basharat; University of Nottingham School of Health Sciences, |
| <b>Primary Subject Heading</b>: | Health services research |
| Secondary Subject Heading: | Obstetrics and gynaecology |
| Keywords: | Immigrant women, Maternity care, United Kingdom, Systematic review, Narrative synthesis |
| | |

SCHOLARONE™
Manuscripts

# Interventions that improve maternity care for immigrant women in the United Kingdom (UK): protocol for a narrative synthesis systematic review

*Gina Marie Awoko Higginbottom,[1] Catrin Evans,[2] Myfanwy Morgan,[3] Kuldip Kaur Bharj,[4] Jeanette Eldridge,[5] Basharat Hussain[6]*

**Abstract**

**Introduction:**

A quarter of all births in the UK are to mothers born outside the UK. There is also evidence that immigrant women have higher maternal and infant death rates and of inequalities in the provision and uptake of maternity services/birth centres. The topic is of great significance to the National Health Service (NHS) because of directives that address inequalities and the changing patterns of migration to the UK. Our main question for the review is "what interventions exist that are specifically focused on improving maternity care for immigrant women in the UK"? The primary objective of this synthesis is to generate new interpretations of research findings and other literature. Secondly, the synthesis will provide substantive base to guide developments and implementation of maternity services/birth centres which are acceptable and effective for immigrant women in the UK.

**Methods and analysis:**

We are using a narrative synthesis (NS) approach to identify, appraise and synthesise empirical evidence focused on access and interventions that improve maternity care/birth centres for immigrant women in the UK. The inclusion criteria include: publication date 1990 to present, English language, empirical research and findings, study participants live in the United Kingdom, study participants are immigrant women, is related to maternity care/birth centres access or interventions or experiences of maternity.

To ensure robustness of the NS, the methodological quality of key literature will be appraised using the Center for Evidence Based Management tools and review confidence with CERQual. Two reviewers will independently screen articles, extract relevant data. We will synthesise evidence exploring relationships within and between studies using a range of tools.

**Dissemination:**

Dissemination plan include: an e-workshop for policy makers, collaborative practitioner workshops, YouTube video and APP, scientific papers and conference presentations.

**Study registration number:** PROSPERO 2015:CRD42015023605

For peer review only



For peer review only - http://bmjopen.bmj.com/site/about/guidelines.xhtml

**Box-I: Strengths and limitations of this study**

- Our systematic review uses a narrative synthesis (NS) approach to identify, appraise and synthesise reports on empirical research focused on access and interventions that improve maternity care/ birth centres for immigrant women.
- A NS a) facilitates understanding and acknowledges the broader influences of theoretical and contextual variables such as ethnicity, socioeconomic status, and geographical location; b) enables understanding of the shaping of differences between reported outcomes as a result of differing study designs and childbearing populations; c) provides results that enable the development and implementation of maternity services/birth centres and interventions.
- We will review qualitative, quantitative and mixed-methods research evidence.
- The review will use the "four dimensions of access" theoretical framework in the healthcare context.
- This is not a meta-synthesis thus limiting our access to the raw data.

## INTRODUCTION

The UK is in a period of super-diversity [1] with more diverse populations accessing maternity care.[1-4] Super-diversity "is distinguished by a dynamic interplay of variables among an increased number of new, small and scattered, multiple- origin, transnationally connected, socio-economically differentiated and legally stratified immigrants" [5 p:1024] who started arriving in the UK after 1990. It has been argued that current level of super-diversity in the UK has resulted in a huge challenge for understanding and meeting individualised healthcare needs.[5]

Facilitating the provision of appropriate health care for immigrant populations in the UK is critical for maximizing health potential and well-being.[2 3 6-8] Without culturally appropriate and safe care delivery, a negative trajectory of events may occur that range from simple miscommunication to life-threatening incidents with serious consequences in maternity.[9] For example, immigrant women are over-represented in mortality statistics particularly in maternal and perinatal mortality.[10 11] Whilst recent reviews have focused on specific maternity care aspects [6-8] they have ignored a comprehensive conceptualisation of access  as opposed to Gulliford et al,[12] and failed to embrace current super-diversity.[1] Reconfiguration and design of maternity care/birth centres in the UK requires integration of all these aspects.



For peer review only - http://bmjopen.bmj.com/site/about/guidelines.xhtml

Our review uses Gulliford theory of access.[12] This framework of access is related to healthcare and has four dimensions (Box-II).

**Box-II: Gulliford theory of access in healthcare**

i. Service availability
ii. Utilisation of services and barriers to access, which includes personal, financial and organisational barriers
iii. Relevance, effectiveness and access
iv. Equity and access.

These four dimensions are extremely pertinent in respect of providing a theoretical lens in order to explore the experience of immigrant women. We use this [12] theory because primacy is given to the notion of equity of access as the most significant dimension. The NHS seeks to provide a universal and egalitarian service consequently this might be the most profound and significant dimension of access. Our review provides the opportunity for a nuanced and comprehensive exploration of the four facets of access.

Gulliford theoretical framework on access interfaces with NICE[13] research recommendations on access and models of service provision. NICE regards some immigrant women as having complex social factors. It notes that pregnant women, who are recent migrants, asylum seekers or refugees, or who have difficulty reading or speaking English, may not make full use of antenatal care services. This may be because of unfamiliarity with the health service or because they find it hard to communicate with healthcare staff. The NICE guidance suggests that *"healthcare professionals should help support these women's uptake of antenatal care services by, using a varied of means to communicate with women, telling women about antenatal care services and how to use them, and undertaking training in the specific needs of women in these groups" (p.16).* The guidance further recommends that healthcare staff be given specific training in the meeting the needs of the group.

Our aim is to undertake a narrative synthesis of a wide range of empirical literature including synthesis of grey literature to provide stakeholders with perspectives on access and maternity care/birth centres interventions (NHS & non NHS) directed at immigrant women in the UK.

Our objectives for this synthesis include:



For peer review only - http://bmjopen.bmj.com/site/about/guidelines.xhtml

- To identify, appraise and synthesize qualitative, quantitative and mixed-methods empirical studies on the topic,
- To identify, appraise and synthesize grey literature and non-empirical reports,
- To identify additional knowledge users and mechanisms of knowledge transfer (KT)
- To share our findings through strategic end-of-grant KT.

Ultimately, we will establish the current knowledge base and generate important recommendations for future policy and practice/programming thus mapping out pathways to health equity.

To reach our aim we are using a project advisory group (PAG), initiated during the establishment of the review questions and early planning for dissemination, and as planned for the entire project duration. The group will be active partner in the research process and engage in all steps in the review.

**METHODS AND ANALYSIS**

We will follow a narrative synthesis approach to systematic review. According to Popay et al NS is "an approach to the systematic review and synthesis of findings from multiple studies that relies primarily on the use of words and text to summarise and explain the findings of the synthesis".[14] The emphasis is on an interpretive synthesis of the narrative findings of research rather than on a meta-analysis of the data. NS will allow us to embrace cross-disciplinary and methodologically pluralistic research to map access and interventions in maternity care/birth centres. The major findings of this synthesis will then be used to explain how and why maternity care/birth centres interventions focused on immigrant women have been implemented and how these interventions address access and inequalities experienced by immigrant women. Our preliminary scoping review (devised by a research librarian) has helped identify a range of quantitative and qualitative studies focused on access and interventions which enabled us to determine that a synthesis does not yet exist with a specific focus on our review question.

NS comprises four elements: a) Development of a theory of how the intervention works, why it works, and for whom b) Development of a preliminary synthesis of the findings of the included studies c) Exploration of the relationships in the data d) Assessment of the robustness of the synthesis. These elements are not exclusive and the synthesis takes an iterative approach. In each element, a variety of tools and techniques may be used depending on the nature of the research evidence.

**Eligibility criteria**



For peer review only - http://bmjopen.bmj.com/site/about/guidelines.xhtml

For peer review only

**Study characteristics**

This review will review qualitative, quantitative and mixed methods research evidence. Therefore, no study will be excluded based on its type. All research evidences published in English language from 1990 to present will be eligible for inclusion in the review. We are focusing 1990 as the immigration patterns in the UK changed 1990 onward. Prior to 1990, immigration to the UK was predominately from India, Pakistan, Bangladesh, and Caribbean. The review will include peer reviewed and grey literature.

**Population**

We will review empirical and grey literature that report on maternity care/birth centres for immigrant women in the UK. We define an immigrant as a person who is born outside the UK, has foreign citizenship, and have moved to the UK to stay temporarily (at least a year) or has intention to settle for the long-term.[15] Therefore, economic migrants (both skilled and unskilled), refugees, asylum seekers, students and illegal immigrants' population will be included.[16] Literature on British-born Black and Minority Ethnic (BAME) participants would be ineligible for inclusion in the review.

**Intervention**

By intervention we mean "a combination of program elements or strategies designed to produce behaviour changes or improve health status among individuals or an entire population".[17] The interventions that we plan to review are those that immigrants participate in during the antenatal, intra-partum or postnatal period in healthcare or social or community setting. Therefore only interventions relevant to pregnancy and the postpartum period (up to 12 months post-delivery) will be reviewed. Interventions must be specifically focused on immigrant women and those aimed at the general population will be excluded.

**Context**

Maternity health services/birth centres related interventions that are offered in the United Kingdom. Interventions focused on immigrants in any setting i.e. hospital and community will be included.

**Outcomes**

The primary outcome from this project would be a narrative synthesis of literature relating to maternity care/birth centres for immigrant women in the UK which would be publish in high impact journal e.g. Journal of Health Services Research and Policy, Social Science and Medicine, and open-access journals (e.g. BMC Pregnancy & Childbirth, BMJ Open Access). Key findings and recommendations would be shared with relevant stake



For peer review only - http://bmjopen.bmj.com/site/about/guidelines.xhtml

holders through online workshop, seminars and meetings. Regarding secondary outcome, this project would provide an important basis to guide development and implementation of maternity care/birth centres services which are acceptable and effective for immigrant women in the UK.

**Data sources and search strategy**

The following literature databases will be searched for relevant articles published between 01 January 1990 and 30 June 2017; the searching and retrieval of results will be completed between February-June 2017. The quoted date range for each database and preferred host system are recorded below:

- Medline (1946 – present, Ovid),
- Embase (1980 – present, Ovid),
- PsycINFO (1806 – present, Ovid),
- HMIC (1979 – latest monthly update, Ovid),
- MIDIRS (1971 – latest monthly update, Ovid),
- CINAHL (to present, EBSCOHost),
- ASSIA (1976 – present, ProQuest),
- POPline (http://www.popline.org),
- Web of Science (1900 – present),
- Scopus (1970 – present)

An experienced research librarian will construct the detailed search strategies for each database and conduct the searches after review by the entire team. The Ovid MEDLINE search strategy will be adapted for each database so that relevant controlled vocabulary, searching techniques, and keywords are used (Appendices-Table-I). Reference lists of included studies will be reviewed for relevant citations and additional hand-searches will be undertaken in major relevant journal e.g. J Immigr Health) and as published by topical research groups. BH and JE will implement the search strategy independently (independent double screening), and meet to review any discrepancies or dissonance. Ambiguous papers will be reviewed by the entire team.

The search for grey literature will include thesis repositories (e.g. ProQuest Dissertations & Theses), Google Scholar, internet-based searches, citation searches, clinical trials registers, research funders' listings of research projects, reviews of reference lists. Grey literature will be screened for relevance and categorised using the National Information Centre on Health Services Research system.[18]



For peer review only - http://bmjopen.bmj.com/site/about/guidelines.xhtml

**Data management**

The review will use Endnote Bibliographic management software to manage the searches. All search hits will be directly imported into the Endnote and duplicates will be removed by the two reviewers independently.

**Selection process**

We will use the PRISMA flow chart (fig.I) to document the review steps.[19] We will employ a three-stage process: (I) screening; (II) preliminary categorization (III) retrieval, final selection and final categorization, employing independent double screening. The screening of peer reviewed articles include screening of titles, screening of abstracts with the attached screening tool (Appendices-Table-II). This tool will be piloted by two reviewers with the first 10% of the database 'hits'.

Grey literature items will be screened for relevance and categorized using the categories of grey literature established by the National Information Centre on Health Services Research and Health Care Technology at the National Library of Medicine (Appendices-Table-III).[18]

The two reviewers will independently extract data from each included study and any disagreement will be resolved through discussion, if needed involving the third reviewer to reach consensus. Data will be sought on variable such as which migrant group(s) the intervention(s) is focused, length of migration, type of intervention, setting of intervention (Hospital/community), duration of intervention, intervention making agency (NHS, social care, voluntary group).

Four researchers experienced in numerous forms of systematic reviewer having undertaken formal training and conducted subsequent funded reviews [20-22] will be involved in data extraction (GH, CE, KB, BH). A relevancy appraisal will be undertaken by first reviewing the title and abstract (GH,CE,KB,BH) and then retrieving potentially relevant articles for further assessment to meet our inclusion criteria and with entire team deciding final inclusion. The methodological quality of key literature will be appraised using the Center for Evidence Based (CEBMa) Management tools (e.g. critical appraisal of a survey, critical appraisal of a qualitative study, critical appraisal of a case study)[23] and for assessment of confidence in the NS review findings CERQUAL.[24] We are using critical appraisal tools of CEBMa as it has a full range of tools as opposed to Critical Appraisal Skills Programme (CASP)e.g. CASP does not have a tool for survey research.



For peer review only - http://bmjopen.bmj.com/site/about/guidelines.xhtml

**Missing data**

Attempts will be made to contact study authors via email to obtain clarification if data are incomplete in the study report and we will allow a delay of six weeks to receive a response following two email attempts.

**Quality Appraisal** We plan to assess quality and the robustness of the synthesis through following ways:

*I) Weight of evidence*: Popay et al. [14] identifies the weight of evidence approach as a tool that can be used to assess the robustness of the synthesis results.

*II) Critical reflection:* Popay et al.[14] recommend that a summary discussion of the synthesis should be provided and includes the following:

a) Methodology of the synthesis – especially focusing on the limitations and their possible impact on the results,

b) Evidence used, in terms of quality, reliability, validity and generalizability (for quantitative papers), with an emphasis on possible sources of bias (for qualitative papers we will apply Lincoln and Guba's principles of confirmability, transferability, credibility, and dependability,[25]

c) Assumptions made,

d) Discrepancies and uncertainties identified, and how discrepancies were dealt with,

e) Areas where the evidence is weak or non-existent, possible areas for future research, and finally,

f) A discussion of the evidence presented will consider the 'thick' and 'thin' evidence [20] and comment on similarities and/or differences between evidence (GH, MM, CE, KB, BH). 'Thick' evidence offers adequate detail to achieve an understanding of the phenomenon of our interest, whereas 'thin' evidence is unable to provide appropriate detail to give an understanding of the phenomenon.[14]

**Strategy for data synthesis**

We have planned a narrative (descriptive) synthesis for this review for all types of studies. Patterns emerging from the textual descriptions and cross-literature comparisons will allow us to identify the factors that affect maternity interventions and the implementation of maternity services/birth centres. These factors will be synthesized into major themes regarding barriers and enablers that shape interventions related to immigrant women and maternity care/birth centres services. We will use conceptual and thematic analysis using a range of clustering and networking tools. In addition to tabulation, we will use analytical software for further grouping (i.e atlas.ti7 by ATLAS.ti GmbH, Berlin, Germany).



For peer review only - http://bmjopen.bmj.com/site/about/guidelines.xhtml

## DISSEMINATION

A multi-level knowledge translation (KT) plan will be used so that key messages are strategically delivered to key stakeholders. We will facilitate an online workshop with key-decision makers/stakeholders to ensure policy relevant messages and utilise widely accessible technology (social networking, webinars) to maximize impact.

The research team involves individuals who are engaged in community/hospital-based health services with immigrant women. KT will continue through community meetings with families, women's groups, and workshops related to refugee/immigrant health. Health and social care knowledge users will be invited to attend community-based initiatives e.g. seminars/workshops.

Contribution to theory and practice will occur via publication of findings in high-impact international journals, such as J Immigr Minor Health, Sociology of Health and Illness, Journal of Health Services Research and Policy, and Social Science and Medicine and open-access journals (e.g. BMC Pregnancy & Childbirth, BMJ Open Access).

## Author affiliations

[1]Prof. Gina Marie Awoko Higginbottom
The Mary Seacole Professor of Ethnicity and Community Health
School of Health Sciences
The University of Nottingham
B Floor, South Block Link, Queen's Medical Centre
Nottingham, NG7 2HA
t: +44 (0) 115 82 30991
e-mail: gina.higginbottom@nottingham.ac.uk

[2]Dr Catrin Evans, Associate Professor
School of Health Sciences, University of Nottingham, UK
A Floor, South Block Link, Queen's Medical Centre
Nottingham, NG7 2HA
e-mail: catrin.evans@nottingham.ac.uk

[3]Professor Myfanwy Morgan
Emeritus and Visiting Professor
Institute of Pharmaceutical Science
King's College London, UK
e-mail: myfanwy.morgan@kcl.ac.uk

[4]Dr Kuldip Kaur Bharj (KB) OBE, Associate Professor in Midwifery,
Faculty of Medicine and Health
School of Healthcare, University of Leeds, UK
e-mail: k.k.bharj@leeds.ac.uk

[5]Jeanette Eldridge
Senior Research Librarian, Medicine & Health Sciences



For peer review only - http://bmjopen.bmj.com/site/about/guidelines.xhtml

Research and Learning Services,
University of Nottingham, UK
e-mail: jeanette.eldridge@nottingham.ac.uk

[6]Dr Basharat Hussain **(Corresponding author)**

Senior Research Fellow
School of Health Sciences
The University of Nottingham
B Floor, South Block Link, Queen's Medical Centre
Nottingham, NG7 2HA
e-mail: basharat.hussain@nottingham.ac.uk

**Contributors**:

GH conceptualised the review and drafted the original grant proposal with CE, MM and KB. JE developed and implemented the scoping review strategy. BH drafted the manuscript for publication with input from other team members. All authors approved the final version of the protocol for publication.

**Funding:**

This work is supported by the UK National Institute for Health Research (NIHR) Health Services and Delivery Research Programme by grant number (HS&DR-15/55/03). Along with funding, NIHR also contributed in peer reviewing the funding proposal containing the review protocol. Any amendments (if required) in the protocol would be done in consultation with NIHR.

**Sponsor:** Department of Health (DoH)- NIHR (543808)

**Competing interests**: None.

**REFERENCES**

1. Phillimore J. Migrant maternity in an era of superdiversity: New migrants' access to, and experience of, antenatal care in the West Midlands, UK. *Social Science & Medicine* 2016;148:152-59. doi: 10.1016/j.socscimed.2015.11.030
2. Fisher J, Hinchliff S. Immigrant women's perceptions of their maternity care: a review of the literature part 2. *The practising midwife* 2013;16(2):32-34.
3. Shortall C, McMorran J, Taylor K, et al. Experiences of Pregnant Migrant Women receiving Ante/Peri and Postnatal Care in the UK: A Doctors of the World Report on the Experiences of attendees at their London Drop-In Clinic, 2015.
4. Garcia R, Ali N, Papadopoulos C, et al. Specific antenatal interventions for Black, Asian and Minority Ethnic (BAME) pregnant women at high risk of poor birth outcomes in the United Kingdom: a scoping review. *BMC pregnancy and childbirth* 2015;15(1):226.
5. Vertovec S. Super-diversity and its implications. *Ethnic and racial studies* 2007;30(6):1024-54.
6. Coast E, Jones E, Portela A, et al. Maternity care services and culture: a systematic global mapping of interventions. *PLoS One* 2014;9(9):e108130.
7. Oakley L, Gray R, Kurinczuk J, et al. P14 Interventions to increase the early initiation of antenatal care in socially disadvantaged and vulnerable women: a systematic



For peer review only - http://bmjopen.bmj.com/site/about/guidelines.xhtml

review. *Journal of Epidemiology and Community Health* 2010;64(Suppl 1):A39-A39.
8. Hollowell J, Oakley L, Vigurs C, et al. Increasing the early initiation of antenatal care by Black and Minority Ethnic women in the United Kingdom: a systematic review and mixed methods synthesis of women's views and the literature on intervention effectiveness. *Final Report University of Oxford* 2012
9. Higginbottom GM, Safipour J, Yohani S, et al. An ethnographic study of communication challenges in maternity care for immigrant women in rural Alberta. *Midwifery* 2015;31(2):297-304.
10. Knight M, Kurinczuk JJ, Spark P, et al. Inequalities in maternal health: national cohort study of ethnic variation in severe maternal morbidities. *BMJ* 2009;338:b542.
11. Manktelow B, Smith L, Seaton S, et al. MBRRACE-UK Perinatal Mortality Surveillance Report, UK Perinatal Deaths for Births from January to December 2014. *The Infant Mortality and Morbidity Studies, Department of Health Sciences, University of Leicester, Leicester* 2016
12. Gulliford M, Figueroa-Munoz J, Morgan M, et al. What does' access to health care'mean? *Journal of health services research & policy* 2002;7(3):186-88.
13. National Institute for Health and Clinical Excellence (NICE). Pregnancy and complex social factors: A model for service provision for pregnant women with complex social factors. In: guidelines N, ed., 2010.
14. Popay J, Roberts H, Sowden A, et al. Guidance on the conduct of narrative synthesis in systematic reviews. *A product from the ESRC methods programme Version* 2006;1:b92.
15. Anderson B, Blinder S. Who counts as a migrant? Definitions and their consequences. *Briefing, The Migration Observatory at the University of Oxford* 2017
16. Urquia ML, Gagnon AJ. Glossary: migration and health. *Journal of Epidemiology and Community Health* 2011:jech. 2010.109405.
17. Intervention MICA : Building healthy communities. What is an intervention 2017 [Available from: http://health.mo.gov/data/interventionmica/ accessed 15th February 2017.
18. National Information Center on Health Services Research and Health Care Technology (NICHSR). Health Services Research and Health Policy Grey Literature Project: Summary Report 2006 [Available from: https://www.nlm.nih.gov/nichsr/greylitreport_06.html accessed 15th February 2017.
19. Moher D, Liberati A, Tetzlaff J, et al. Preferred reporting items for systematic reviews and meta-analyses: the PRISMA statement. *PLoS med* 2009;6(7):e1000097.
20. Higginbottom GM, Hadziabdic E, Yohani S, et al. Immigrant women's experience of maternity services in Canada: A meta-ethnography. *Midwifery* 2014;30(5):544-59.
21. Higginbottom GM, Morgan M, Alexandre M, et al. Immigrant women's experiences of maternity-care services in Canada: a systematic review using a narrative synthesis. *Syst Rev* 2015;4(1):13-17.
22. Higginbottom G, Bell AS, Arsenault J, et al. An integrative review of experiences of maternity services for immigrant women in Canada. *Diversity & Equality in Health and Care* 2012
23. Center for Evidence-Based Management. Critical Appraisal Tools & Questionnaires 2017 [Available from: https://www.cebma.org/ accessed 15th February 2017.
24. Lewin S, Glenton C, Munthe-Kaas H, et al. Using qualitative evidence in decision making for health and social interventions: an approach to assess confidence in findings from qualitative evidence syntheses (GRADE-CERQual). *PLoS Med* 2015;12(10):e1001895.
25. Lincoln YS, Guba EG. Naturalistic inquiry: Sage 1985.



For peer review only - http://bmjopen.bmj.com/site/about/guidelines.xhtml

For peer review only



For peer review only - http://bmjopen.bmj.com/site/about/guidelines.xhtml

**Figure Legends**

Figure I: PRISMA flow chart showing review steps

Table I: Detailed Search strategy to be used

Table II: Title and Abstract Screening tool

Table III: Inclusion of Grey Literature using established categories by AcademyHealth.

For peer review only



For peer review only - http://bmjopen.bmj.com/site/about/guidelines.xhtml

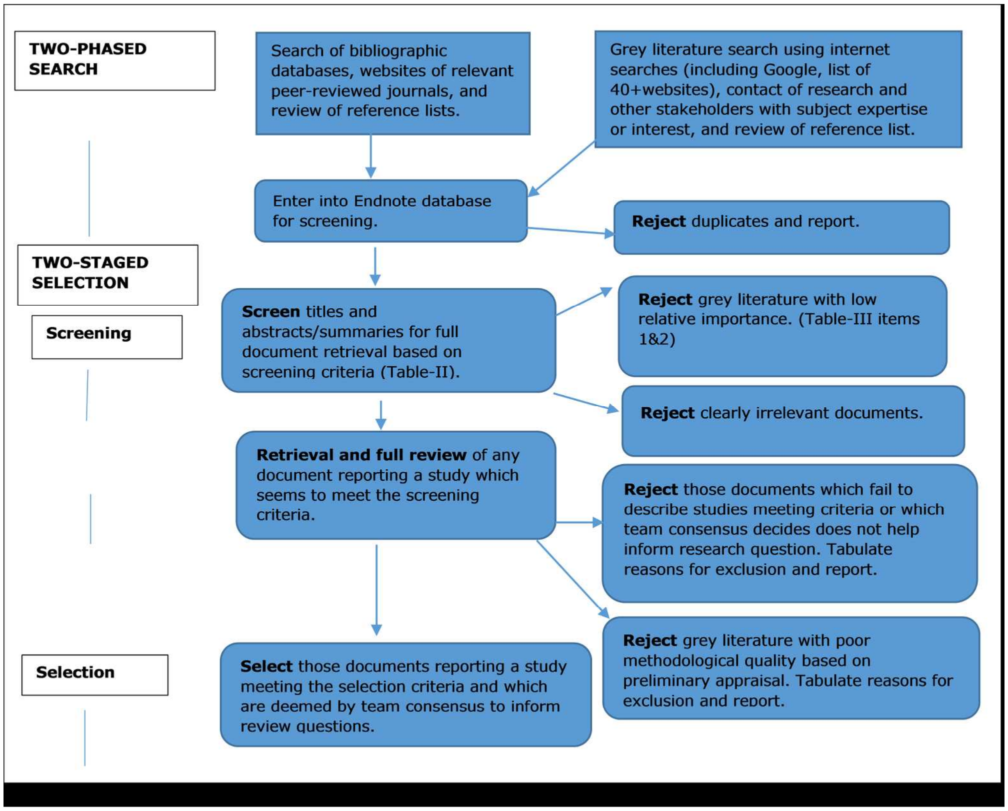


Review steps

198x158mm (300 x 300 DPI)

**Appendices:**

**Table-I**

**10/03/2017**

**Ovid MEDLINE(R) In-Process & Other Non-Indexed Citations and Ovid MEDLINE(R) 1946 to Present**

| | |
|---|---|
| 1 | exp Maternal Health Services/ or exp Postnatal Care/ or exp Preconception Care/ or exp Prenatal Care/ or exp Perinatal Care/ or exp Infant Care/ or exp Midwifery/ or exp Obstetrics/ or exp Obstetric Nursing/ |
| 2 | exp maternal welfare/ or exp maternal care/ or exp maternal child health care/ or exp newborn care/ or exp prepregnancy care/ |
| 3 | exp General Practitioners/ or exp Primary Health Care/ or exp Family Health/ or exp Community Health Nursing/ |
| 4 | (((maternal or child* or baby or babies or fetus* or fetal* or embryo* or obstetric*) adj3 (health* or nurs* or care or service*)) or (midwif* or midwiv*)).ti,ab. |
| 5 | ((birth* or matern* or mother* or pregnan* or childbearing or child-bearing or prenatal or pre-natal or postnatal or post-natal or perinatal or peri-natal or preconception or pre-conception or antenatal or ante-natal or postpartum or puerperium) adj3 (health* or nurs* or care or service*)).ti,ab. |
| 6 | exp Health Services Accessibility/ or exp Healthcare Disparities/ or exp Health Services/ |
| 7 | (3 or 6) and (matern* or child* or baby or babies or fetus* or fetal* or embryo* or obstetric* or birth* or mother* or pregnan* or childbearing or child-bearing or prenatal or pre-natal or postnatal or post-natal or perinatal or peri-natal or preconception or pre-conception or antenatal or ante-natal or postpartum or puerperium).ti,ab. |
| 8 | 1 or 2 or 4 or 5 or 7 |
| 9 | ("use" or access* or utili* or consum* or block* or hurdl* or barrier* or hindr* or hinder* or obstacle* or exclu* or discrimin* or disparit* or disproportion* or inequal* or unequal* or inadequat* or insuffic* or stratif* or limit* or lack* or unreliab* or poor* or poverty* or depriv* or disadvantag* or insecur* or insensit* or status* or entitl* or uninform* or ill-inform* or benefit* or interven* or deliver* or effective* or cost effective*).ti,ab. |
| 10 | (3 or 6) and 9 |
| 11 | Emigrants and Immigrants/ or Refugees/ or "Transients and Migrants"/ or "Emigration and Immigration"/ |
| 12 | (((established or long-term or "first generation*" or new* or recent* or current*) adj3 (migrant* or migrat* or immigrant* or immigrat* or emigrant* or emigrat* or emigre* or expat* or (ex adj pat*) or transient* or alien*)) or newcomer* or (new adj comer*) or incomer* or (in adj comer*) or ((international or overseas or foreign) adj2 (student* or employee* or worker*))).ti,ab. |
| 13 | (refugee* or (asylum adj seek*) or asylee* or (refused adj3 (asylum* or refugee*)) or (displaced adj person*) or exile* or (new adj arrival) or (country adj2 (birth or origin)) or transnational*).ti,ab. |
| 14 | (foreigner* or (foreign adj (born or citizen* or national* or origin*)) or (non adj (citizen* or native*)) or ((adoptive or naturali#ed) adj (citizen* or resident*)) or overstay* or trafficked or "spousal migrant*").ti,ab. |
| 15 | ("non-UK-born" or "born outside the UK" or "length of residence in the UK" or (("not lawful*" or "not legal*" or unlawful* or illegal* or unauthori#ed* or "not authori#ed" or uncertain or insecure or illegal or legal or legitimate* or permit* or visa* or irregular* or refused or undocumented) adj3 (residen* or student* or worker* or employee* or unemployed* or immigrant* or imigrat* or migrant* or migrat*))).ti,ab. |
| 16 | exp Ethnic Groups/ or (ethnic* or ethno* or race or racial*).ti,ab. |
| 17 | exp african continental ancestry group/ or exp asian continental ancestry group/ |
| 18 | exp Vulnerable Populations/ or ((vulnerab* or disadvantag* or minorit*) adj3 (individ* or person* or people* or population* or communit* or group*)).ti,ab. |

For peer review only

| | |
|---|---|
| 19 | ("Black and Minority Ethnic" or "Black & Minority ethnic" or BME or BAME or african caribbean* or afro caribbean* or black african* or (west adj (indies or indian*))).ti,ab. |
| 20 | (south asia* or afghan* or bangladesh* or bengal* or bhutan* or india* or maldiv* or nepal* or pakistan* or sri lanka*).ti,ab. |
| 21 | 11 or 12 or 13 or 14 or 15 or 16 or 17 or 18 or 19 or 20 |
| 22 | 8 and 10 and 21 |
| | |

**Table-II: Preliminary Screening Tool**

| **Citation** ________________________________________<br>**Criteria for selection** | Yes | No | Can't say |
|---|---|---|---|
| 1. Publication date 1990 to present | | | |
| 2. English language | | | |
| 3. Empirical research and findings | | | |
| 4. Study participants live in the United Kingdom | | | |
| 5. Study participants are immigrant women (where there is mixed sample of immigrant and non-immigrant women each paper must have findings specific to immigrant women) | | | |
| 6. Is related to maternity care/birth centres access or interventions or experiences of maternity | | | |

❖ An article needs to meet 1-6 to be screened.

**Table-III: National Information Centre on Health Services Research and Health Care Technology (NICHSR). Health Services Research and Health Policy Grey Literature Project: Summary Report 2006**

| 5 | 4 | 3 | 2 | 1 |
|---|---|---|---|---|
| • Working papers<br>• Testimony<br>• Committee reports<br>• Conference proceedings | • Data evaluations<br>• Foundation reports<br>• Government reports<br>• Grantee Publications<br>• Non-commercially published conf. papers<br>• Reports<br>• Special reports | • Speeches<br>• Annual Reports<br>• Presentations<br>• Grantee Reports<br>• Webcasts<br>• Theses<br>• Technical specifications & standards | • Newsletters<br>• Bibliographies<br>• Bulletins<br>• PowerPoint Presentations<br>• Foundation financial statements | • Pamphlets<br>• Protocols<br>• Guidelines<br>• Poster Sessions<br>• Meeting agendas<br>• Translations |

For peer review only

Grey literature items falling in columns three to five will be given consideration for inclusion as these demonstrate relatively higher significance to policy makers and related users of grey literature.

For peer review only

**PRISMA-P (Preferred Reporting Items for Systematic review and Meta-Analysis Protocols) 2015 checklist: recommended items to address in a systematic review protocol***

| Section and topic | Item No | Checklist item |
|---|---|---|
| **ADMINISTRATIVE INFORMATION** | | |
| Title: | | |
| Identification | 1a | Identify the report as a protocol of a systematic review **(01)** |
| Update | 1b | If the protocol is for an update of a previous systematic review, identify as such **(It is new protocol)** |
| Registration | 2 | If registered, provide the name of the registry (such as PROSPERO) and registration number **(PROSPERO 2015:CRD42015023605)** |
| Authors: | | |
| Contact | 3a | Provide name, institutional affiliation, e-mail address of all protocol authors; provide physical mailing address of corresponding author **(9-10)** |
| Contributions | 3b | Describe contributions of protocol authors and identify the guarantor of the review **(10)** |
| Amendments | 4 | If the protocol represents an amendment of a previously completed or published protocol, identify as such and list changes; otherwise, state plan for documenting important protocol amendments **(10)** |
| Support: | | |
| Sources | 5a | Indicate sources of financial or other support for the review **(10)** |
| Sponsor | 5b | Provide name for the review funder and/or sponsor **(10)** |
| Role of sponsor or funder | 5c | Describe roles of funder(s), sponsor(s), and/or institution(s), if any, in developing the protocol **(10)** |
| **INTRODUCTION** | | |
| Rationale | 6 | Describe the rationale for the review in the context of what is already known **(2-3)** |
| Objectives | 7 | Provide an explicit statement of the question(s) the review will address with reference to participants, interventions, comparators, and outcomes (PICO) **(01, 3-4)** |
| **METHODS** | | |
| Eligibility criteria | 8 | Specify the study characteristics (such as PICO, study design, setting, time frame) and report characteristics (such as years considered, language, publication status) to be used as criteria for eligibility for the review **(05)** |
| Information sources | 9 | Describe all intended information sources (such as electronic databases, contact with study authors, trial registers or other grey literature sources) with planned dates of coverage **(6)** |
| Search strategy | 10 | Present draft of search strategy to be used for at least one electronic database, including planned limits, such that it could be repeated (**6 attached** ) |
| Study records: | | |

For peer review only

| | | |
|---|---|---|
| Data management | 11a | Describe the mechanism(s) that will be used to manage records and data throughout the review **(6)** |
| Selection process | 11b | State the process that will be used for selecting studies (such as two independent reviewers) through each phase of the review (that is, screening, eligibility and inclusion in meta-analysis) **(7-8)** |
| Data collection process | 11c | Describe planned method of extracting data from reports (such as piloting forms, done independently, in duplicate), any processes for obtaining and confirming data from investigators **(7-8)** |
| Data items | 12 | List and define all variables for which data will be sought (such as PICO items, funding sources), any pre-planned data assumptions and simplifications **(7-8)** |
| Outcomes and prioritization | 13 | List and define all outcomes for which data will be sought, including prioritization of main and additional outcomes, with rationale **(5)** |
| Risk of bias in individual studies | 14 | Describe anticipated methods for assessing risk of bias of individual studies, including whether this will be done at the outcome or study level, or both; state how this information will be used in data synthesis **(8)** |
| Data synthesis | 15a | Describe criteria under which study data will be quantitatively synthesised **(N/A)** |
| | 15b | If data are appropriate for quantitative synthesis, describe planned summary measures, methods of handling data and methods of combining data from studies, including any planned exploration of consistency (such as $I^2$, Kendall's $\tau$) **(N/A)** |
| | 15c | Describe any proposed additional analyses (such as sensitivity or subgroup analyses, meta-regression) **(N/A)** |
| | 15d | If quantitative synthesis is not appropriate, describe the type of summary planned **(9)** |
| Meta-bias(es) | 16 | Specify any planned assessment of meta-bias(es) (such as publication bias across studies, selective reporting within studies) |
| Confidence in cumulative evidence | 17 | Describe how the strength of the body of evidence will be assessed (such as GRADE) **(8)** |

*** It is strongly recommended that this checklist be read in conjunction with the PRISMA-P Explanation and Elaboration (cite when available) for important clarification on the items. Amendments to a review protocol should be tracked and dated.** **The copyright for PRISMA-P (including checklist) is held by the PRISMA-P Group and is distributed under a Creative Commons Attribution Licence 4.0.**

*From: Shamseer L, Moher D, Clarke M, Ghersi D, Liberati A, Petticrew M, Shekelle P, Stewart L, PRISMA-P Group. Preferred reporting items for systematic review and meta-analysis protocols (PRISMA-P) 2015: elaboration and explanation. BMJ. 2015 Jan 2;349(jan02 1):g7647.*

For peer review only
